# Chemistry, Meteorology, and the Function of Digestion, Considered with Reference to Natural Theology

**Published:** 1846-01

**Authors:** 


### Art. VII.

*Chemistry, Meteorology, and the Function of Digestion, considered with Reference to Natural Theology.* By William Prout, m.d. f.r.s., Fellow of the Royal College of Physicians. *London,* 1845. Third Edition, 8vo, pp. 515.

The high merit of this work, and the respect we entertain for its learned author, induce us to make a departure in its favour from our usual practice; and to avail ourselves of the opportunity afforded by the appearance of this new edition, to bring it under the special notice of our readers, although in its original form it made its appearance eleven years ago,—one year before the commencement of our critical labours. Coming out as one of the series of Bridgewater Treatises, the origin and plan of which are doubtless familiar to all our readers, Dr. Prout's work enjoyed at the time an extensive popularity, and came to a second edition in the course of a few months. We remember hearing the opinion expressed at the time by a competent judge, that this popularity would be more durable than that of most of the other works of the series,—Dr. Prout being in many respects in advance of the general state of knowledge on his science, whilst some of the other authors were scarcely on a level with it. We rejoice to see this opinion borne out by the appearance of a third edition at this distant date, indicating as it does the continued, though perhaps not very rapid, sale of the work. A considerable amount of new matter has been introduced, by an enlargement of the page, without any augmentation in the bulk of the volume; and this is for the most part of much value. We think that Dr. Prout has done wisely in restricting his additions within moderate limits, and in not entering upon the field of controversy; and the modest apology which he makes in his Preface must disarm criticism in regard to what may be deemed errors of omission. "The author is still conscious of many imperfections, arising in no small degree from his professional avocations. The varied and difficult nature of the points to be investigated required more undivided attention than he has been able to bestow."

The work commences with a chapter entitled, "Of the leading Argument of Natural Theology; that Design, or the Adaptation of Means to an End, exists in Nature." From this we shall extract a passage, which presents an admirable summary of the fundamental points of the argument:

"Our belief, then, in the agency of an intelligent Creator, is founded—

"On our recognition of the identity of the effects produced in external nature, with effects produced by ourselves; from which identity of effect we immediately infer identity of purpose,—the *existence of design* without reference to a designer.

"On our consciousness that the purpose effected by us proceeded from ourselves, the designers; whence we conclude that the design manifested in external nature must have had a like origin,—that the manifestation of design is demonstrative of the existence of a designer.

"On the pervading character of the design shown among the objects of nature, in which design man recognizes the *creation* of the objects designed, and is thus led to infer the existence of a *Creator*. Now the faculty of reason, which enables man to recognize the Creator of the objects around him, enables him to recognize in that Creator the *Creator of himself and of his faculties*. In reasoning, therefore, from his own acts, to those of the Creator of the universe, though conscious that he is reasoning from the finite to the Infinite, from weakness to Almighty Power; yet, when he reflects from whom he has derived his faculty of reason, man feels assured that his own reasoning, when it coincides with the reasoning evinced by his Creator, *can be no other than the same*. Nor, founded as that assurance is on the constitution of the human mind, can that assurance be impugned, without impugning Him by whom the human mind has been so constituted.

"Thus the argument of design, though not based on necessity, in the strict sense of the term, is of a validity equal to that of our knowledge of the existence of, and of our connexion with, an external world." (p. 7.)

In the second chapter are considered "The rank of Chemistry as a Science; and the Argument of Design with reference to Chemistry." The argument, as treated by Paley, is thus summed up by Dr. Prout:

"Chemistry is a department of knowledge founded solely on experience, for the phenomena of which we can assign no reason. But though the intimate nature of chemical changes be unknown to us, we see them manifestly directed to certain ends; hence, as things directed to certain ends, where the whole of the intermediate phenomena can be traced and understood, always imply design; we naturally infer design in chemical changes obviously so directed, even although we may not be able to understand their intimate nature." (p. 17.)

We are much disposed to agree with Dr. Prout, however, in thinking that the very circumstance of the mysterious nature of the operations of chemistry, gives them an impressive interest, which is wanting in those classes of phenomena which are better understood. We witness the utmost order and regularity, as is evidenced in the "Law of definite proportions;" but we cannot account either for this general uniformity, or for the limitations in the combining powers of different substances, for want of some more intimate knowledge of the forces that are concerned, and of their mode of operation. As to the bearing of this deficiency on the argument from design, Dr. Prout remarks as follows:

"Obvious mechanism, though well suited to display the intelligence and design of the Contriver, is not always so well adapted for arresting the attention of the observer, its very obviousness in some measure depriving it of its interest. But when we see the same Contriver, besides the most beautiful and complicated mechanism, employ other means utterly above *our* comprehension, though evidently most familiar to *Him*, the employment of these means is not only calculated to arrest our attention more forcibly, but at the same time to impress us with more exalted notions of His wisdom and power." (p. 18.)

It is far from our intention to go through the work chapter by chapter, but we wish to offer a few remarks on the general nature of the argument from design, for which this will be the most fitting place, as they naturally connect themselves with the closing passages of this section. The first of these has reference to the possible denial of the reality of the changes termed chemical:

"Perhaps one of the most striking arguments in favour of the reality of chemical changes may be deduced from the subserviency to them of those mechanical contrivances and operations everywhere existing in organized beings. At least half the mechanism in a living animal is subservient to the chemical changes constantly going on in it, and necessary to its existence. Take, for instance, the circulation of the blood: what a complicated apparatus is here employed for the simple purpose of exposing the blood to the action of the air in the lungs, in order that it may there undergo some chemical change. Now, surely no one can reasonably doubt that this chemical change is as much a reality as the mechanism by which the chemical change has been accomplished; and if one chemical change be admitted to be a reality, must not all the others be equally real?" (p. 19.)

There is a class of objectors to the argument from design, who affect to believe that all the beautiful arrangements and adaptations which we witness in creation arise from what they term "the necessary and eternal laws of nature." Such persons are either pantheists, making gods of these "laws of nature;" or, if they recognize a Creator at all, it is by some other mental process. They are thus addressed by Dr. Prout:

"If there be any one who denies the existence of design, and sees nothing in all the more obvious arrangements and order around him but the necessary result of what he chooses to denominate 'the laws of nature,' let him calmly and deliberately consider the facts brought forward in the following pages; and if he can witness unconvinced all the numerous instances of prospective arrangement clearly made with reference to things not yet in existence; all the beautiful adjustments and adaptations of noxious and conflicting elements, most wonderfully conspiring together for good; and lastly, the subversion of even his favourite 'laws of nature' themselves, when a particular purpose requires it: if, we say, he can witness all these things, and still remain incredulous of the evidences of design, his mind must be most singularly constituted, and apparently beyond the reach of conviction." (p. 20.)

The fundamental error of such objectors is their assumption that those *human* expressions of a certain order and regularity in the phenomena of the universe, to which the term "law of nature" is commonly given, are the necessary and inherent properties of matter. This error we have exposed on a recent occasion, (vol. XIX. pp. 160-61.) We shall add to our quotations on this subject one taken from the recapitulation of the argument, as applied to the phenomena of organic chemistry, with which the work concludes:

"The adaptations of mechanical arrangements in the structure of organized beings to the pre-existing chemical properties of matter, afford also an evidence of design, not less impressive than unequivocal. The most determined sceptic cannot assert that there is any *necessary* relation, or indeed any relation whatever, between the mechanical arrangements and the chemical properties to which they administer. There is no reason why the chemical changes of organization should result from the mechanical arrangements by which they are accomplished; neither is there the slightest reason why the mechanical arrangements in the formation of organized beings should lead to the chemical changes of which they are the instruments. From what cause, then, arose the association of the chemical changes with the mechanical arrangements? How were the chemical operations of digestion and respiration brought into union with the mechanical apparatus of digestion, and with the circulating system? The coexistence of things so entirely dissimilar, and having no kind of mutual relation, can be explained only on the supposition that *a will exists somewhere,* and also *a power to execute that will.* The existence is thus unavoidably acknowleged of a Being who, knowing every pre-existing chemical property of matter, and willing to direct these chemical properties to a specific object, has contrived for that purpose an apparatus admirably fitted to attain His object. Such is the explanation—the only possible explanation, of the subservience of mechanism to chemistry, in the processes of organic life. And what is this explanation but our *argument of design,* in terms that seem absolutely irresistible?" (p. 488.)

There is perhaps no more remarkable instance to be found in the world of organization, of that *general adaptation* which seems to us to afford by far the most satisfactory foundation for theological arguments, than is presented in the concurrence here adverted to by Dr. Prout. Take, for example, the process of respiration as performed in the higher animals; and enumerate the number of operations all tending towards the same purpose, all interwoven as it were in one system, and yet all essentially independent. For the simple purpose of bringing the blood into relation with the atmosphere, and of permitting the chemical changes which necessarily result from that communication, what a multitude of mechanical provisions are required. First, an organ of most remarkable and elaborate construction, within which the blood and the air may act upon each other; this organ consisting of innumerable chambers of extreme minuteness, yet these so connected by a series of branching passages with the main outlet, that a continual interchange of their gaseous contents may be effected; whilst the walls of these chambers are covered with a capillary network, closer than that which is to be found in any other part of the body, so as almost to expose a continuous film of blood to the air within, and so communicating with afferent and efferent trunks, that a continuous supply of unaerated blood may be conveyed to every part, whilst that which has undergone the required change shall be immediately removed. And if such be the provisions required in the lungs themselves, how vastly are they surpassed in variety and complexity by those which keep the lungs in play; alternately filling their cavities with the pure air inhaled from the atmosphere, and withdrawing their contents when tainted by the poisonous exhalations of the blood, and maintaining through their capillaries a continuous flow of blood, whose rate of movement is so precisely adjusted as to permit the vivifying and restoring influence of the air to be fully exerted, and yet with the least possible delay! What an almost countless multitude of provisions and operations are destined to this one set of purposes! What an elaborate adaptation of the bony parietes of the chest to the formation of a cavity, which shall be perfectly closed and yet capable of constantly alternating dilatation and contraction by forces acting upon its exterior,—which shall afford adequate protection to the delicate organs within, at the same time that it serves for the attachment and support of the anterior limbs,—and which shall yet be so flexible as to accommodate itself to every movement of the trunk—so light that its weight is never felt! What an apparatus of muscles, each consisting of countless fasciculi of fibres, every one having its appropriate action, neither repeating another; some being in constant and regular operation; whilst others are reserved for those more energetic movements, which are sometimes required by peculiar conditions of the system, or which serve to compensate in a degree for the occasional imperfect action of other parts of the apparatus! How wonderful is the connexion of these various muscles with the nervous system, which keeps them in constant alternating movement without any effort of the will, even without a thought, during sleep, in utter unconsciousness,—which regulates their actions as to frequency and intensity, so as to supply a quantity of air exactly proportioned to the amount of blood to be aerated, and the degree of purification which it requires,—and which is yet placed under such a degree of subservience to the will, as to allow their movements to be adapted to the needs of the vocal apparatus, and thus to contribute to that function which, more than all others, binds together the different individuals of a race by mutual intercommunication! And how inefficient would be all this mechanism, how useless all its well-devised operations, if it were not made to concur with another wonderful organ; by whose ceaseless pulsations the dark tide of blood returning impure from the body, is propelled to the lungs; whose flood-gates open wide to receive it again, as it returns purified and renovated by exposure to the atmosphere; and whose vigorous contractions force the vivifying current into the remotest corners of the body!

We have sketched but the broad outline of this argument, as suggested by our author's indication; it would occupy us too long to fill it up with anything like minuteness of detail; and we must leave it to the sagacity of our readers to trace out the various subsidiary provisions, which are requisite for the due operation of the machine, whose chief parts and principal actions we have thus passed under review. Now to our own minds, the existence of such a provision, or series of provisions, in a single animal or group of animals,—however elaborate the design might appear, and however perfect might be its operation,—would have little or no theological importance. It *might* be regarded as an instance of successful construction, without imputing *design* or *intentional adaptation of means to ends.* The inference that would be founded upon it, by a person on the outlook for examples of such design, might be met by the objection that we see only *one* out of *many* possible combinations of organs; that supposing these to have been made without any design, according to the ordinary laws of chances *some one* might prove to be fitted for a continued existence on the surface of the globe whilst all the rest perished long since; and that consequently such an isolated example *proved* nothing whatever as to the capacity of the original Designer. Such an objection, it seems to us, could not be easily rebutted; for every one is familiar with cases in which pure *accident* has accomplished a purpose better than any *design.* Thus, when a painter had been labouring unsuccessfully for many hours to produce the appearance of foam at a horse's mouth, and in a fit of vexation and despair threw his sponge, charged with a variety of colours, against the picture, the very effect was produced which he had been in vain exhausting his powers to attain, and this by the merest combination of chances, which might never occur again. The sponge *happened* to be charged with colours, in the precise amounts and proportions requisite to produce the effect; and it *happened* to be flung by the painter in such a manner as to alight on the precise spot where these colours should be deposited, and to deposit just the amount of colour which gave the desired appearance. It would be scarcely possible for any one, struck with the exactness of the representation, but ignorant of the means by which it had been brought about, to avoid the conclusion that the most elaborate design had been exercised in producing the effect; especially when he considered that this was but a small portion of a work, on which he *knew* that a designing mind had been employed. And yet he would be completely mistaken. The same liability to error, as it appears to us, pervades every theological argument erected upon *single* adaptations of means to ends in the organized creation; and this more especially, since we have no right to assume that a designing mind has been exerted about it at all,—that such a mind has been at work upon any part, being the very thing to be proved.

Now we will suppose that our painter, struck with the result of this casualty, had adopted the method in other cases, and had executed a number of other pictures, in all of which (after many trials and failures, it may be) the same effect was successfully repeated. It is obvious that any person who witnessed this result would be much more entitled to suppose it *intentional*, than he would be from the single case first cited. And further, we will suppose that, instead of repeating precisely the *same* effect, the painter so varied the colours in his sponge and the manner in which he threw it, as to produce variations in the effect, which should be adapted to represent correctly the different appearances that would be appropriate to a *variety* of animals; it is obvious that an observer who knew nothing of the method, and only witnessed the result, would be still more justified in regarding it as proceeding from a *designed* adaptation of means to ends, and would indeed feel it almost impossible to resist the conclusion that it was so, even when informed of the *casual* nature of the first or single adaptation.

Just so, as it seems to us, must the most complete sceptic, if not so prejudiced as to be *determined* to resist conviction, yield to the cumulative argument from design, whilst he may question the supposed proof drawn from isolated cases. Thus, to revert to the subject on which we just now touched; whilst the existence of the most perfect adaptation between the mechanical and chemical operations of a single organized body, in the function of respiration, *proves* nothing in regard to the intention or design of this adaptation, the evidence accumulates when we observe the same adaptation existing, the same plan followed out, in other groups of living beings; and it attains its greatest force, when we contemplate the variation of the methods which are employed in different groups to compass the same end, these variations being in every instance adapted most exactly to the general wants of the system. Thus, whilst the description we have given of the respiratory apparatus applies chiefly to mammals, we find that apparatus extended in birds through the entire system, and so arranged that, whilst really constructed upon a lower type, it shall impart a greater amount of aeration to the blood, and at the same time confer buoyancy upon the body. In reptiles, on the other hand, a still further degradation of type,—shown in the small amount of subdivision of the lungs,—is accompanied by a very low amount of aeration; which is conformable to the feeble circulation, sluggish habits, and low temperature of that group. Tracing the air-breathing apparatus, again, through the articulated series, we find a gradual transition from the concentrated lung-like sacculi of the spider tribe,—whose lining membrane is folded like the leaves of a book, so as to expose a large amount of surface in a small space,—to the extended tracheal system of insects, penetrating into every part of the body, supplying the deficiency of circulatory power by the universal diffusion of the air that it conveys, and ministering to an amount of aeration which, like the muscular energy of these animals, surpasses in proportional degree that of all other tribes; and thence, through the more limited tracheal apparatus of the myriapods, to the simple air-sacs of the terrestrial worms, which present the lowest form of organs for atmospheric respiration. We might follow out the leading forms of the apparatus for aquatic respiration,—commencing with fishes, descending through the mollusca and aquatic articulata to the radiated classes,—in the same manner; and might show in detail the beautiful uniformity of the general plan, combined with an endless variety of modifications, adapted to the general habits and conditions of the animal. And we might then revert to geology for proof that we do not find these adaptations, because the animals that present them *happen* to be fitted to live and to continue their race in the circumstances in which we find them; for were it so, we ought to find for one such casual instance of adaptation an almost infinite number of failures: whilst the fact is, that the remains of extinct animals which the progress of research brings to light, all exhibit the same plan, with modifications adapted to the circumstances of their existence; and that, so far from these having come into existence by anything like that "fortuitous concourse of atoms" which was once a favorite method of accounting for the existing state of things, and having then immediately perished because incapable of continuing their existence,—we have ample evidence that they lived and died, eat, drank, and propagated their kind, through countless successions of ages, until some change in the condition of their dwelling-place rendered it no longer fit for their habitation, and subjected them to the general law of extinction, which had already cleared the way, by its operation on previous races, for *their* appearance on the scene.

This is an outline of the course of argument which, as it seems to us, is required to build up the science of natural theology upon its true basis. We would not discard the ordinary inferences drawn from isolated cases, for they are well fitted to make an impression on minds, which cannot appreciate the grander and more general views we have indicated, and which do not perceive the slenderness of the support on which they rest; and they aid, by their accumulation, in forming the extended and secure foundation which is required for solidity. It must be obvious, we think, that when phenomena of any kind are viewed as the necessary results of general laws, or, to speak in language which we deem more correct, as consistent parts of a uniform plan,—their mutual harmony and adaptation becomes much more striking, than when it is regarded as expressly contrived in each individual case, in accordance with the ends to be attained, as they present themselves. For the one act implies a comprehensive acquaintance with *all* the results of the plan laid down, and a mutual adaptation of the different parts of the plan, in the first instance; whilst from the other might be inferred a limited conception of those results, requiring to be corrected when the machine should be put in motion.

Now, we cannot but think that those who planned and executed the Bridgewater Treatises have in general erred in dwelling too much upon the *particular*, and too little upon the *general*; in accumulating individual instances of design, and in directing but little attention in comparison towards the general plan, of which those instances are but isolated examples. Thus we have a whole treatise devoted to the Mechanism of the Hand, by an anatomist who, with all his accomplishments, was unable to comprehend the philosophy of his science, and who went out of his way to sneer at those who are engaged in its development. So again we have the subject of Animal and Vegetable Physiology separated from Geology; thereby putting it out of the power of either writer to give a comprehensive view of the organized creation, and of what is known of the plan on which it has been developed; since in such a view, the *past* and the *present* must be equally embraced.

Although the subjects of Dr. Prout's Treatise appear to be somewhat heterogeneous, yet they lead not unnaturally into each other; and we are disposed to think that the plan of treatment is on the whole more adapted to its purpose, than is that of most of the other Treatises. After the introductory chapters, to which we have already referred, Dr. Prout proceeds to those most general and fundamental ideas on which the science of chemistry rests; and gives a luminous view of the present state of our knowledge of the "molecular actions of matter," connected by an ingenious hypothesis of his own. The facts, however, are kept sufficiently clear from the hypothesis, for any intelligent reader to separate them with facility; and although we do not find that hypothesis altogether satisfactory, we are disposed to think that the author has done wisely in introducing it, as showing the mode in which general principles may be applied. No argument illustrative of design is founded upon the supposed molecular arrangements he has given; his purpose being rather to show that the *invisible* operations of chemistry are at least as wonderful as the *visible* operations of mechanism, and are, like them, capable of being reduced under simple and determinate principles. "The foregoing attempt," he says in conclusion, "to connect physical molecular phenomena is acknowledged to be very imperfect. Enough, however, has been said to draw the attention of those who are interested in the subject. Another age will follow out these or some nearly allied principles; and the physical phenomena of the microcosm will thus become as much a matter of mathematical inquiry and proof, as the physical phenomena of macrocosm or universe now are." (p. 163.) This division of the work closes with an admirable chapter, comprising general reflections and arguments founded on the preceding details; from this we shall select some passages in illustration. After noticing, as an example of the adjustment between the conditions of the different materials of our globe, the mutual adaptation of the properties of water and the temperature of the earth, Dr. Prout thus continues :

"If we do not admit of this adjustment, we must suppose that the whole has been the result of chance, or of some other unintelligent principle; and if water had been the only case in which such adaptations were apparent, the supposition of chance might perhaps be received, at least it would be difficult to prove the contrary. But when we see similar happy adjustments in every object around us,—in the different elements of the air we breathe, in the soil we tread upon, in all the varieties of rocks composing the solid crust of our globe; not one of which could have been more happily contrived for the purposes they fulfil, nor indeed be scarcely conceived to exist otherwise than as they are, without destruction to the whole of the present arrangements,—when we see all these things and duly reflect on them, it becomes absolutely impossible to suppose that so much happy adjustment, so much apparent intelligence, so much, in short, of what the veriest sceptic, under other circumstances, would have allowed to be evidences of design, can be evidences of anything else than design; or have resulted from any unintelligent cause whatever. Hence we are irresistibly impelled to the only rational conclusions the premises appear to admit of, viz., that all these happy adjustments and adaptations which we see in nature, are really and truly what they appear to be,—so many evidences of design; and, consequently, that the whole have sprung from the will of an intelligent and omnipotent Creator" (p. 167.)

As the mutual balancing of the perturbations of the solar system, producing the stability or equilibrium of the whole, affords the most comprehensive foundation for the argument from design in the arrangement of the planetary bodies, so do we think that Dr. Prout appeals to what the reflecting mind must admit to be the most comprehensive form of his portion of the argument, when he rests it on the equilibrium of the several distinct agencies of nature :

"Amidst all the endless diversity of property, and all the changes constantly going on in the world around us, we cannot avoid being struck with the general tendency of the whole, to a *state of repose* or *equilibrium.* Moreover, this tendency to equilibrium is not confined to the ponderable elements, but prevails also, in the same remarkable degree, among the imponderable agencies, heat and light, which, as we have seen, cannot be anywhere long retained in a state of excess, on account of their natural disposition to acquire a certain state of equilibrium, depending generally upon the place of the earth in the solar system. Now, the formation of this state of equilibrium and its preservation, may be considered as the results of those wonderful adjustments among the qualities and quantities of bodies above alluded to; the qualities being such as to neutralize each other's activity, while the quantities are so apportioned as to leave one or two only predominant. It is further to be observed that this state of equilibrium is not absolutely *fixed;* such an unyielding condition would be not less incompatible with the present order of things, than a condition of unlimited change. All things therefore are so adjusted, that slight deviations or oscillations about the neutral point of rest or equilibrium take place, and are even necessary, as the world is at present constituted; though these changes are bounded within very narrow limits, and greater deviations would instantly prove fatal to the whole." (p. 174.)

"Throughout nature, the exigences and incongruities necessarily arising from the arrangements we have been considering, have given occasion for the display of the most astonishing wisdom and power. And instead of that jarring and clashing, which might have been expected from so many conflicting elements, the qualities and quantities of those elements have, upon the whole, been so wonderfully adjusted to each other, that they neutralize and balance each other's evils; and the general result has been that all have finally settled down together, into that harmonious state of equilibrium before alluded to, so admirably adapted for the existence of organic life." (p. 180.)

We have said that we consider the subject of meteorology as not unnaturally connecting itself with that of chemistry, in such a general survey as that here taken by Dr. Prout; and the transition from one to the other becomes still more obvious, when it is made through the views we have just quoted. For whilst it is the business of the geologist to trace out the history of these various alternating periods of disturbance and of equilibrium or quiescence, which have contributed to produce the present condition of the globe,—and to demonstrate that to these convulsions and changes we owe all that boundless variety of sea and land, of mountain and plain, of hill and valley; all that endless admixture of rocks, of strata, and of soils, so essential to the existence of the present order of things; without which the world would have been a mass of crystals, or one dreary monotonous void, altogether unfit for the support of the present races of organized beings, and precluding the existence of man,—it belongs to the meteorologist more especially to consider the globe in its *present* condition of equilibrium, and the means by which this state of equilibrium is maintained; in particular, to point out the influences of heat, light, and electricity, and of the energies allied to them; to study the laws of the distribution and change of these wonderful agents, in the production of climate; to trace, in short, the effects of heat, light, and electricity, on the earth, the ocean, and the atmosphere; and all the infinite variety of phenomena dependent upon them. This is the method in which Dr. Prout has considered the subject; and we can recommend the chapters on meteorology as containing an admirable summary of those general operations of chemical agents upon the surface of our globe, which are concerned in producing the endless varieties of climate, and in thereby adapting it to the residence of almost innumerable tribes of living beings; the existence of a large number of which would be incompatible alike with any greater uniformity, and with any wider diversity, in its conditions.

The concluding book is not so much a limited account of the function of digestion, which its title seems to imply, as a general sketch of the chemistry of organization, and of the various processes by which alimentary substances are assimilated to, and become component parts of, a living body. At the time of the first publication of this Treatise, this sketch was decidedly in advance of the general state of knowledge on the subject; and many of the views contained in it were as novel as they now are familiar. The author may justly claim the credit of having contributed largely to the direction of inquiry towards these objects; and when the labours of Dumas and Liebig and their followers, are quoted as having built up the present fabric of organic chemistry, the foundation which had been laid by our countryman should not be forgotten. And it would be well if his clearly-drawn distinctions between what chemistry *may*, and what it *cannot*, effect for physiology, were more constantly kept in view. To those chemists who imagine that the employment of the apparatus for ultimate analysis is to revolutionize the science of physiology, we would give it as a problem to be solved,—what light can be thrown by chemistry upon that simplest and most fundamental of all vital operations, the growth of a *cell* from a germ, its gradual development, its production of the germs of new cells in its interior, its operation upon the surrounding elements, so as to combine them into the materials for its own fabric and for the germs of its successors, whilst at the same time it fills its cavity with compounds of a totally new and distinct character, and finally its death, by which its contents are set free,—the cell-germs to go through the same process in their turn, and the other substances to serve, it may be, as materials for some purpose, to all appearance totally unconnected, in the economy of nature? The chemist may ascertain the number of atoms of carbon, hydrogen, oxygen, and nitrogen, which are combined in the substance of the cell-wall, in the reproductive germs, and in the contained products of cell-action. He may ascertain the sources of these in the carbonic acid, the ammonia, and the moisture, which the cell derives from the atmosphere. And when he has done all this, what more can he effect? Or how does this information give any clue to the mysterious properties of the primordial cell-germ,—properties by which it not only operates itself, but gives rise to an unlimited extent of similar operation; properties which are so far peculiar to itself, that no combinations which the chemist can effect can ever exhibit the like, and which are derivable only from a pre-existent being of the same character; properties which are at least as far removed from those which give rise to the physical and chemical operations of matter, as those are from each other? Surely there can be no reasonable expectation that physics and chemistry can ever give any account of these actions, which are rightly denominated vital, as being peculiar to *living* bodies; and the utmost that can be expected in regard to them is that, when the laws of molecular agency are better understood, they may be included with others in some higher and more general expression common to all.

Having said so much in recommendation of Dr. Prout's Treatise, we feel called on to express our complete discordance with his views in one important particular; and it is rather singular that the full development of these views, in a form which almost gives them (to our minds at least) the character of a *reductio ad absurdum*, is to be found only in the present edition, a mere sketch of them being all that was contained in the preceding. In the third chapter, entitled "Of Matter and of Physical Agents in general, and of their mutual relations," we meet with the following passages:

"That matter and the power of moving matter exist, both within our own corporeal frames as well as in the world around us, we cannot doubt, without doubting our own existence. By evidence equally irresistible we are impelled to the belief that matter, and power of moving matter, though everywhere conjointly existent and exerted, must be essentially different. In other words, that matter does not move in virtue of any property necessarily belonging to it as matter, but that matter was first created and set in motion by the Deity when he formed the universe; and that the motion then originally imparted to it is still retained, and will continue to be retained by matter, 'till time shall be no more.'" (p. 22.)

"Of the positive or abstract nature of matter and of power, any more than of the positive and abstract nature of the Deity himself, we know absolutely nothing. . . . . Taking for granted that matter and power exist, and are different, we assume their relationship to each other to be that of *passion* and *agency*. According to this assumption, matter is entirely passive; while the forces by which it is moved are, as it were, personified, i. e., supposed to be wielded by subordinate and delegated existences, capable of exerting physical force according to certain laws, and within certain limits, prescribed by the Deity; of whose will, therefore, they are the immediate executors. In other words, to use the language of Paley, this assumption regards physical agency as exerted by 'subordinate beings, to whom the Deity, after having laid down certain laws and furnished certain materials, has delegated the task of drawing forth a creation.'" (p. 23.)

Dr. Prout is perfectly correct in designating this notion as an *assumption;* and its baseless character becomes so evident upon the slightest consideration, that we must confess our astonishment that it should have been adopted and sustained by a mind like our author's. We believe that it is now universally admitted by the profoundest logicians and physical philosophers of our day, that *we can form no idea of matter except through its properties;* and that the *forces* or *powers*, by which the various material phenomena are produced, are nothing else than *those properties in action.* Thus our most simple and general idea of matter depends upon its *resistance* to our touch; and that resistance is due to nothing else than the operation of the mutual attraction, subsisting among the particles of the substance touched,—and varying in its relative amount in substances of different kinds, or in the same substance according as it exists in the solid, fluid, or gaseous state. Now this mutual attraction of its particles, more or less balanced by a mutual repulsion, is essential to our very idea of matter; so completely so, in fact, that it has been argued by one of the profoundest philosophers of our day, with a degree of vigour which must impress if it does not convince, that we gain nothing in our explanation of the molecular actions of matter, by regarding the centres of the spheres of attraction and repulsion as being occupied by material particles or atoms; but that if we regard these centres as mathematical points, we shall be at least as well able to explain those phenomena of attraction and repulsion which give rise to our fundamental notions of matter.* According to this view, then, *matter* is nothing but *force.* We do not profess to adopt it; but we unhesitatingly affirm that we can form no idea of matter but by its forces, and that the assumption that *matter* and *force* are distinct, and are respectively characterized by *passion* and *agency,* is not only unnecessary but highly illogical. The idea, too, of "delegated existences," appears to us to involve so many difficulties, as to be completely untenable; and we cannot but regard it as altogether a fiction of the imagination. If all matter, with its properties, originally came into existence at the Creator's *fiat,* why should we suppose any "delegated existence" to have the charge of regulating its subsequent operations, which are nothing else than the original properties in action, or the *continued expressions* of that same Will which at first called the universe into being? To suppose a "delegated existence" necessary to superintend particular classes of operations, is, in our apprehension, to bring down the attributes of the Deity to a human standard, by limiting the exercise of Omniscience and Omnipotence to the general superintendence, and committing the details to subordinates.

But it is when this doctrine is applied to the organized creation, that its objectionable character seems to us most palpable; for the "delegated existences," here termed "organic agents," are regarded by Dr. Prout as possessing the rank of "*independent intelligent agents,* superior

* See Prof. Faraday on Electric Conduction and the Nature of Matter, in Taylor's Philosophical Magazine, 1844.

to, and possessing the power of directing and controlling, the common forces of matter, so as to cause these forces to associate material elements into the organized, instead of the crystallized condition. (p. 399.) We cannot but think that he is quite mistaken in affirming that this hypothesis, "variously modified, is not only the most ancient, but with some reserve and under different disguises, is that which is most generally adopted by physiologists to the present time." For although the notion of some kind of existence has been very prevalent under various names—the last, and the one in general use, being that of "vital principle"—we know of few if any philosophers, who have been bold enough to affirm that the "organic agent" is "a conscious being possessing knowledge, will, and power." (p. 399.) In fact Dr. Prout himself in his former editions, and in his Gulstonian Lectures, characterized the "organic agent" as "an ultimate principle, endowed by the Creator with *a faculty little short of intelligence*;" and it is only in the present edition, that he has gone to his present extreme. The inconsistencies of his former hypotheses were most ably exposed by Mr. Robertson in his 'Critical Remarks on certain recently-published Opinions concerning Life and Mind,' (London, 1836;) and we extract from that publication a passage which anticipates the progress of Dr. Prout's views. "Did Dr. Prout unequivocally allow that the organic agent possesses intelligence, the inquirer would be easily satisfied; for from analogy he would be led to regard this agent as an order of being most amazing in the variety and compass of its powers, surpassing the human intellect in an infinitely greater degree than the latter surpasses the intellect of the worm or zoophyte. And the longer he pursued the comparison between the powers and resources of his own mind and those of this agent, the more irresistibly would he feel the conviction stealing upon him, that Dr. Prout's 'organic agent' is no other than that great Being, who alone, in the language of Scripture, is 'wonderful in counsel and excellent in working.'" It is, indeed, fully admitted by Dr. Prout, that the active intelligence, which can alone explain organization, is vested in the great Source of all knowledge and of all power; but he considers that the idea of mediate agency is in general most in accordance with natural operations. Now, we have already pointed out, that the hypothesis is alike cumbersome and illogical in regard to ordinary physical phenomena; and we cannot see on what firmer basis it rests in regard to those of a vital character. Fully agreeing with Dr. Prout "that matter and material forces when properly directed are fully adequate to form organized bodies;" and that "material forces fulfil the will of the Creator in organic processes, without any knowledge or will of their own," (p. 401); we must confess our inability to see any ground for the assumption, or to detect what is gained by the hypothesis, that the will of the Creator is not directly exerted upon matter in the construction of organized bodies, but employs a mediate agent. Dr. Prout maintains that "the hypothesis of mediate agency is not in any way derogatory to the Deity, but the reverse; inasmuch as the creation of an intelligent agent capable of accomplishing his purposes conveys to us a more exalted notion of his power, than the accomplishment of his purpose by himself." We cannot say that this appears to us in the same light; and we shall illustrate our view of it by a simile. Suppose a general to be in command of a large army, every component individual of which is perfectly under his control. If he had the means of so operating *immediately* upon the minds of every soldier, as to cause him to execute his will without any intervening command, and if he himself possessed the knowledge and skill requsite not only to guide the general evolutions of the *masses* composing the army, but to direct the movements of every *man*, in the most advantageous manner,—would he trouble himself with the intermediate machinery of subordinate generals, colonels, captains, lieutenants, sergeants, and corporals, in order to convey his will and direct the movements of the troops? Or if he should do so, still retaining his absolute power over every individual action, would the function of his officers be anything else than that of mere mouth-pieces? As an army is at present constituted, there is large room for the operation of intelligence in the subordinates or delegated powers; because it is upon them that the carrying-out of the will of the commander into its details entirely rests; and they have not only to choose the best means of doing this, but to modify and accommodate it to circumstances, sometimes adding to it, sometimes (it may be) even acting in opposition to it. But this is the necessary result of the imperfection of the *human* commander, whose knowledge and power are alike limited, and who is compelled to act through the medium of subordinates. To imagine that anything is gained by the interposition of "intelligent agents" between the Deity and the materials on which he operates, is either to set limits to his knowledge and his power, or to give to those agents an office purely nominal. For if they have any of that freedom of choice in their actions, which is possessed to a certain extent by the officers of an army, then the Almighty intelligence is *not* directing the operations of matter in their detail; whilst if that detailed direction has a real existence, they are merely like so many instruments through which the Creator acts, and there is no room for the operation of intelligence in them. Or if it should be argued that they are left free to choose, in ignorance of the will of the Creator, who has nevertheless predetermined what they *shall* choose (which seems to be the position of man,) we answer that this supposition is completely at variance with the fixity and determinateness of the phenomena of organization; which, when properly surveyed, give to us an idea of *law*, or in other words of being the working-out of a definite and uniform plan, fully as much as do those of physics and chemistry. Wherever we see the operations of a *limited intelligence*, as in man and some of the higher animals, there we witness an indefiniteness and want of fixity, to which the operations of the simplest organism could not be safely left.

We shall refrain from urging any further objections to the doctrine in question; not, however, from want of argument, but for want of space; but we shall extract the two passages in which it is carried out to its fullest extent, and leave it to our readers to digest them, if they can. To us they present the hypothesis in a form so utterly unphilosophical, as scarcely to require further disproof.

"Admitting then that the Deity acts through the intervention of delegated existences, and that neither matter nor material forces, nor any assumed *vital* forces can be supposed to possess intelligence; we are driven to the assumption, that this intelligence is vested in the immaterial conscious beings; in other words, that one or more agents possessing knowledge, will, and power, exist in every organized body, from the lowest to the highest. In its lowest grade, the knowledge and operations of such agent may be supposed to be confined to the properties of the material elements adapted for organization, which it has the knowledge to select and the power to form, into an apparatus enabling it to organize other matters, and effect ulterior purposes. Where the operations of this primary organic agent terminate, those of another and higher organic agent may be supposed to begin; which by carrying the general process of organization a step further, adapts the organized material for the operations of a third and superior agent. Thus each new agent may be supposed to possess more or less control over all the agents below itself, and to have the power of appropriating their services, till at length in the combined operations of the whole series of agents at the top of the scale, we reach the perfection of organic existence. (p. 403.)

We cannot understand this passage in any other way, than as implying that there are numerous grades, and an almost infinite individual multiplication, of "organic agents," in every one of the higher organisms. For, as a single cell may be a living being in itself, and may maintain an independent existence, it must necessarily possess its own organic agent; and, by parity of reasoning, since every cell in a complex organism performs its functions in virtue of its own inherent powers, it must be alike furnished with its organic agent—consequently there must be as many distinct intelligent agents in the body of a man, as there are individual cells. This, indeed, we may infer to be Dr. Prout's view, from the following passage:

"The ranks and grades among organic agents are doubtless as numerous as the ranks and grades among organized beings; but organic agents have been, and as far as our present views are concerned, may be classed under three grades. *Organic* agents strictly so called, the immediate fabricators of plants and animals: *animal* organic agents; and the intellectual agent peculiar to man; which properly speaking ought not to be classed with organic agents; since it cannot be shown that the intellectual agent in man has anything to do with the structure of his corporeal frame. In man, as we have said, all these three agents coexist. The organic agent or agents strictly so called, the immediate fabricators of his body, whose intelligent agency consists in wielding material forces, and, by their aid, of organizing matter into the primary condition of irritable cells. Various agents of higher rank, having the capacity of controlling organic agents, and of modelling the primary irritable cells into masses and *forms*, suited to their purposes. And lastly, the intellectual agent, who, capable to a certain extent of influencing all below itself, employs the organized machinery constructed for its use, and suited to its exigences, by the inferior agents, and thus becomes the organized being, man." (p. 406.)

We must confess our inability to discern the essential difference between this figment, and the mythical notions of the ancients respecting their presiding deities or semi-deities, which we presume that Dr. Prout would condemn as absurd. And we think it would not be very difficult to erect just as good an argument in defence of *their* existence, as that which Dr. Prout has put forth as the foundation of his system of organic agents. Thus, why should we not suppose an "intelligent agent" concerned in the selection and arrangement of the particles of silver in a certain metallic solution, so as to develop the *arbor dianæ;* or why should not suppose that Mercury is exercising a *choice* in behalf of the particles of his metal, when they leave one combination and pass into another, by the exercise of what is commonly termed *elective affinity?*

It is but justice, however, to Dr. Prout that we should add—what, indeed our readers may have already inferred from the recent development of this doctrine—that it is essentially independent of the rest of the work, and in no way interferes with its philosophical or theological value, except as constituting an ugly excrescence, from which we should be very glad to see it freed.

